# Prenatal Care and Hypertensive Gestational Syndromes: A Systematic Review

**DOI:** 10.1055/s-0038-1660526

**Published:** 2018-06-20

**Authors:** Gláucya Raquel Souza da Fonsêca Dutra, Laio da Costa Dutra, Gabriela Karine Souza da Fonsêca, Mauro Bezerra do Nascimento Júnior, Eudes Euler de Souza Lucena

**Affiliations:** 1 Department of The Post-Graduation Program In Health and Society, Universidade do Estado do Rio Grande do Norte, Mossoró, RN, Brazil; 2 Department of Odontology, Universidade do Estado da Paraíba, Campina Grande, PB, Brazil; 3 Departament of Nursing, Universidade do Estado do Rio Grande do Norte, Caicó, RN, Brazil; 4 Department of Odontology, Universidade do Estado do Rio Grande do Norte, Caicó, RN, Brazil

**Keywords:** prenatal care, medical assistance, prenatal education, hypertension pregnancy-induced, gestation, assistência pré-natal, assistência médica, educação pré-natal, hipertensão induzida pela gravidez, gestação

## Abstract

**Objective** Evaluate the influence of prenatal care on the occurrence of gestational hypertension.

**Methods** The Web of Science, Scopus, Pubmed, Cochrane and ClinicalTrials electronic databases were searched for articles published between January 1^st^, 2012 and December 31^st^, 2016. No language restrictions were imposed. The following keywords were used: prenatal care, medical assistance, prenatal education, pregnancy-induced hypertension. The preferred reporting items for systematic reviews and meta-analyses (PRISMA) checklist was employed. Two hundred and forty articles were identified during the initial search, but only seven met the inclusion criteria. This systematic review is registered with the international prospective register of systematic reviews (PROSPERO; #CRD42017064103).

**Results** The seven studies had a low risk of bias, with methodological quality scores ranging from six to eight points. Five studies found a positive relationship between prenatal care and pregnancy-induced hypertension, whereas two studies found no significant association between the two variables. The divergence among the studies may have been due to the type of healthcare service at which the study was conducted and the sample size.

**Conclusion** Although the studies analyzed differed with regard to methodological aspects, the findings demonstrate the importance of prenatal care during the gestational period as a prevention and health promotion measure.

## Abstract (pt)

**Resumo:**

**Objetivo** Avaliar a influência da assistência pré-natal no acometimento de síndromes hipertensivas gestacionais.

**Métodos** A revisão buscou artigos publicados nas plataformas eletrônicas de pesquisa Web of Science, Scopus, Pubmed, Cochrane e Clinical Trials, sem restrições de linguagem e com os artigos publicados entre 01/01/2012 e 31/12/2016. Os descritores utilizados foram: assistência pré-natal, assistência médica, educação pré-natal, hipertensão induzida pela gravidez, gestação. Foi utilizado o checklist *preferred reporting items for systematic reviews and meta-analyses* PRISMA. A busca na literatura, de acordo com a estratégia adotada, identificou 240 artigos. Contudo, somente 7 artigos foram selecionados de acordo com os critérios de inclusão. A revisão sistemática foi incluída no registro prospectivo internacional de revisões sistemáticas (PROSPERO, na sigla em inglês; #CRD42017064103).

**Resultados** Cinco estudos encontraram relação positiva entre a assistência pré-Natal e síndromes hipertensivas gestacionais. Dois estudos não encontraram uma associação estatística significativa entre estas duas variáveis. Os sete estudos apresentam um baixo risco de viés, com as pontuações na análise de qualidade variando entre seis e oito. As possíveis diferenças entre os achados podem ser devidas ao momento do diagnóstico das síndromes hipertensiva gestacionais, tipo de serviço onde foi realizada a pesquisa e o tamanho amostral.

**Conclusão** Embora os estudos apresentem diferentes aspectos metodológicos, observou-se a importância da implementação da Assistência Pré-Natal durante o período gestacional, o que atuará como medida de promoção e prevenção em saúde.

## Introduction

Most women experience pregnancy with no complications. Some, however, have characteristics or conditions that can place their health and the health of the fetus at risk. One such condition is pregnancy-induced hypertension (PIH), which is considered a public health problem due to its frequency as well as maternal and perinatal morbidity and mortality, affecting ∼ 10% of pregnancies throughout the world.^1^ ^2^ ^3^

The periodic measurement of blood pressure in pregnant women is essential to the precise diagnosis of hypertension, which can cause serious problems, such as stroke, premature birth or low birth weight.^4^ Pregnancy-induced hypertension occurs when the increase in blood pressure reaches or surpasses 140 × 90 mm Hg. The following classification is used: gestational hypertension, chronic arterial hypertension, chronic hypertension in conjunction with preeclampsia, preeclampsia and eclampsia. The prevalence and incidence of these conditions are quite high in Brazil and vary depending on age group, race, obesity and the presence of associated diseases, such as diabetes and kidney disease.^5^

Despite the vast accumulation of scientific knowledge in recent years, PIH continues to have serious repercussions. Therefore, individualized care is fundamental for early diagnosis and the establishment of interventions to minimize risks to the mother and fetus.^6^ The occurrence of avoidable deaths among pregnant women is associated with insufficient economic, cultural and technological conditions in a given society, making this a serious health problem throughout the world. Such deaths could be avoided if pregnant women had access to quality prenatal care.^7^

Perinatal outcomes are the result of a complex network of biological, socioeconomic and healthcare determinants. Prenatal care can contribute to more favorable situations by enabling the timely detection and treatment of adverse health conditions and the control of risk factors related to complications for the health of the mother and infant.^8^

The aim of the present study was to perform a systematic review of the literature to evaluate the influence of prenatal care on the occurrence of PIH. The following was the research question: Does greater prenatal care diminish the occurrence of PIH?

## Methods

### Selection of Articles

The present systematic review of the literature included cross-sectional, case-control and cohort studies involving patients with PIH. In December 2016, 2 independent reviewers searched 5 electronic databases (Web of Science, Scopus, Pubmed, Cochrane and ClinicalTrials) for articles published between January 1^st^, 2012 and December 31^st^, 2016. No language restrictions were imposed. The following was the search strategy ([Prenatal care OR Medical Assistance OR Prenatal Education] AND [Pregnancy-Induced Hypertension]). The present systematic review is registered with the international prospective register of systematic reviews PROSPERO (#CRD42017064103). The initial online research led to the retrieval of 240 references: 103 in PubMed, 22 in Web of Science, 28 in Cochrane, 84 in Scopus and 3 in ClinicalTrials. Duplicates were removed with the aid of the Reference Manager software, version 12.0.3 (Thomson Reuters, Toronto, ON, Canada), leading to a total of 175 articles, which were analyzed using the eligibility criteria based on readings of the titles and abstracts. Two reviewers underwent a calibration exercise for the application of the eligibility criteria. Following a detailed discussion of the criteria, the reviewers performed independent analyses of a sample of 10% of the abstracts. Interexaminer agreement was determined using the Kappa statistic (K = 0.875).

The inclusion criteria were cross-sectional studies, case-control studies, cohort studies and clinical trials involving prenatal care and PIH, with no restrictions imposed regarding age or language. The exclusion criteria were reviews, clinical cases, editorials, books, abstracts, questionnaire validation studies, studies not addressing PIH, studies published more than 5 years earlier and studies for which data extraction was not possible.

**Fig. 1:**
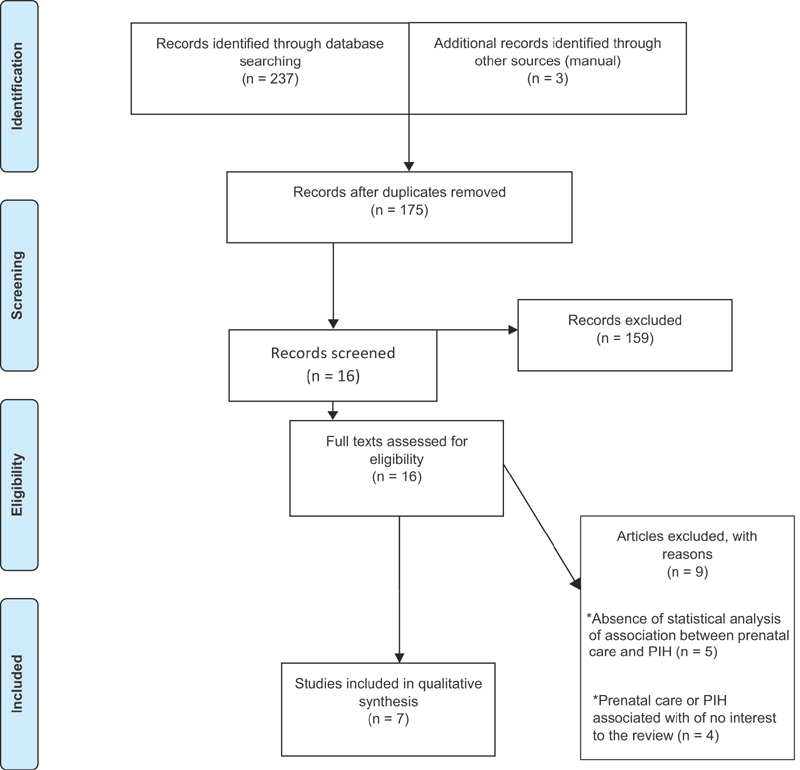
Flowchart of the article selection process.

The application of the eligibility criteria led to the exclusion of 159 articles based on the analysis of the titles and abstracts. Among the 16 articles submitted to full-text analysis, 9 were excluded due to the absence of statistical analysis on the association between prenatal care and PIH or for associating prenatal care or PIH with a variable of no interest to the present systematic review (Fig. 1).

### Data Extraction

Seven studies performed a statistical analysis of the association between PIH and prenatal care and were included in the present systematic review.

### Appraisal of Methodological Quality

Two independent reviewers performed the appraisal of the methodological quality of the studies included in the review using the Newcastle-Ottawa quality assessment scale for case-control and cohort studies.^6^ ^9^ For each article, points were awarded for the presence of each item of the different categories (selection, comparability and exposure/outcome).

### Data Synthesis

The data were grouped based on study design, characteristics of the population and unit of analysis. A narrative synthesis of the data was also performed.

## Results

### Type of Study, Setting and Population Characteristics

The articles selected were three case-control studies, two prospective cohort studies, one retrospective cohort study and a cross-sectional study conducted in Central America, South America, Europe, Asia and Africa. Three studies involved the use of a comparative group classified as a control group. Four studies recruited individuals from hospitals, two recruited individuals from maternity clinics and one study was based on data from a national department of statistics. Patient age ranged from 15 to 72.6 years.

Three studies involved convenience sampling but employed eligibility criteria for the determination of the final sample.^9^ ^10^ ^11^ ^12^ Other studies had population-based samples for the investigation of the proposed factors.^13^ ^14^ ^15^ One study failed to describe how many participants were in the sample.^16^ However, direct contact with the author enabled the determination that the study in question also employed a population-based sample (Table1).

**Table 1:** Studies included in the present review of the association between prenatal care and pregnancy-induced hypertension

Authors, year, country	Type of study	Setting	Sample size	Prevalence; age (years)	Categorization of prenatal care	Categorization of PIH	Statistical analysis	Results	Quality
Sekkarie et al. (2016),^12^ Haiti	Case-control	Rural maternity	689 subjects (67 cases and 622 controls	7.0%; 15–46	No appointments; 1–3 appointments; 4 or more appointments	Preclampsia and eclampsia	Univariate and multivariate	Prenatal care not associated with reduction in risk of PIH: 1–3 appointments (*p* = 0.71; OR = 1.10) and 4 or more appointments (*p* = 0.50; OR = 1.20)	7 (10)
Luo and Ma (2013),^10^ China	Case-control	Tertiary reference hospital	1233 subjects (650 cases and 583 controls)	Prevalence not reported; mean case: 30 + ; control: 28)	Case - mean: M 2.68 ± 6.18 appointments; control - mean: 9.19 ± 4.49 appointments	Preeclampsia	Univariate and multivariate	Preeclampsia associated with fewer prenatal care appointments (*p* < 0.001; OR = 2.04)	7 (10)
Herrera et al. (2014),^16^ Colombia	Prospective cohort	National Department of Statistics	387,000	Private service (pre- eclampsia = 0.4%- 1.4%); Without private service (preeclampsia = 1.4% - 32%); mean: 24.2 ± 6.5 and 25.1 ± 6.9	Implantation of BPSM program	Preeclampsia	Univariate and multivariate	Implantation of BPSM program reduced incidence of pre-eclampsia by 22% (OR = 0.78, 95% CI: 0.67–0.88)	6 (10)
Berhan and Endeshaw (2015),^14^ Ethiopia	Retrospective cohort	University teaching hospitals	1015	612 (60.3%) preeclampsia, 346 (34.1%) eclampsia and 57 (5.6%) other type of PIH; mean: 2.8; range: 15–46.	Prenatal care: yes or no	Chronic hypertension, preeclampsia and eclampsia	Univariate and multivariate	Lack of prenatal care increased risk of PIH (OR 2.3; 95% CI: 1.19 - 4.38).	7 (10)
Assarag et al. (2015),^11^ Morocco	Case-control	Three reference hospitals	299 subjects (80 cases and 219 controls)	Chronic hypertension: 52% of case group and 47% of control group; mean: 29.2 in case group and 28.4 in control group; range: 18–49	Prenatal care: yes or no	Preeclampsia, eclampsia, preeclampsia and eclampsia complicated by hemorrhage	Univariate and multivariate	Lack of prenatal care associated with PIH [OR = 3.97; 95% CI: (1.42–11.09)].	8 (10)
Correia et al. (2015),^15^ Portugal	Transversal	Five public maternities	7,325	3.19%; 55.4% 25–34; 29.7% ≤24; 14.9% ≥ 35	Does not know; 1–2 appointments, 3–6 appointments, 7–9 appointments, ≥10 appointments	Gestational hypertensive disorders (gestational hypertension, preeclampsia and eclampsia)	Univariate and multivariate	Larger or smaller number of appointments in public or private sector did not affect occurrence of PIH (low-risk pregnancy: OR = 1.12; high-risk pregnancy: OR = 0.53)	6 (10)
Männistö et al. (2013),^13^ Finland	Prospective cohort	Community maternity care clinics	10314	17%; mean: 66.7 (62.6 to 72.6)	Prenatal care: ≥ 5 appointments	ISH, IDH, DHP, GH, Preeclampsia /Eclampsia, CH	Univariate and multivariate	Prenatal care associated with ISH, IDH, DHP (*p* < 0.0001); GH (*p* < 0.05); and CH (*p* < 0.001)	7 (10)

Abbreviations: BPSM, biopsychosocial model; CH, chronic hypertension; CI, confidence interval; GH, gestational hypertension; IDH, isolated diastolic hypertension; ISH, isolated systolic hypertension; OR, odd ratio; PIH, pregnancy-induced hypertension; DHP, isolated systolic or diastolic hypertension with proteinuria.

### Categorization of Prenatal Care

Two studies^11^ ^14^ classified prenatal care as present or absent, without specifying the number of appointments. Others^12^ ^13^ ^15^ categorized prenatal care based on the number of appointments. One study^10^ expressed appointment data using mean and standard deviation values for the case and control groups. Another study^17^ used the implantation of a broad prenatal care program based on the biopsychosocial model (BPSM), addressing psychosocial and obstetric factors that promote a reduction in morbidity and mortality rates among pregnant women.^17^ ^18^

#### 3.3 Categorization of PIH

Two studies^11^ ^12^ addressed preeclampsia and eclampsia. Another^9^ used these same categories and included the complication of hemorrhage. Two^10^ ^16^ classified PIH as preeclampsia. One study^14^ specified the conditions as chronic hypertension, preeclampsia and eclampsia. Another study^15^ denominated the conditions as gestational hypertensive disorders, listing gestational hypertension, preeclampsia and eclampsia. Another investigation^15^ used the following categorization: isolated systolic hypertension (ISH), isolated diastolic hypertension (IDH), isolated systolic or diastolic hypertension with proteinuria (DHP), gestational hypertension (GH), preeclampsia/eclampsia and chronic hypertension (CH).

### Statistical Analysis

The studies used prenatal care information as the unit of analysis to investigate the association between prenatal care and PIH. The seven studies^10^ ^11^ ^12^ ^13^ ^14^ ^15^ ^16^ performed univariate, bivariate and multivariate analyses.

#### Relationship between Prenatal Care and Pregnancy-induced Hypertension

The studies reported the following associations: prenatal care with fewer appointments was associated with PIH (*p* < 0.001; OR = 2.04);^10^ a lack of prenatal care increased the risk of PIH (odds ratio [OR] = 2.3 [95% confidence interval [CI]: 1.19 to 4.38]^14^ and OR = 3.97 [95% CI: [1.42 to 11.09]^11^); BPSM reduced the incidence of preeclampsia (OR = 0.78; 95% CI: 0.67 to 0.88);^16^ hypertensive women had a greater number of prenatal care appointments than normotensive women, with significant associations found between ≥ 5 appointments and ISH, IDH, DHP (*p* < 0.0001), GH (*p* < 0.05.) and CH (*p* < 0.001).^13^ Table 1 displays the results of the studies that found no association between PIH and prenatal care. Differences were found in the presentation of effect measures, ORs, *p*-values and CIs.

#### 3.6 Appraisal of Methodological Quality

The methodological quality of the studies analyzed ranged from 6 to 8 points on a 10-point scale (Table 1). The cross-sectional study received a score of 6 points. A total of 4 studies received 7 points, and 1 study received 8 points.

## Discussion

The present systematic review involved a search of multiple databases with no restrictions with regard to language or year of publication. Sixteen articles were preselected for the full-text analysis. A total of 7 met the inclusion criteria and were submitted to an appraisal of methodological quality, receiving scores of 6 to 8 on a 10-point scale.

The researchers described how the cohort and case-control studies occurred, although the eligibility criteria and participant selection methods were not adequately reported. The variables analyzed in the studies, including risk factors and outcome, were well defined. However, none of the studies reported a calibration exercise for the evaluation of the variables.

A case-control study^12^ conducted at a rural maternity clinic in Haiti involving a sample of 689 women (67 in the case group and 622 in the control group) found a 7% prevalence rate of PIH (preeclampsia and eclampsia) among individuals aged 15 to 46 years. The analysis of prenatal care considered the following categories: 0 appointments; 1 to 3 appointments; and 4 or more appointments. Prenatal care was not associated with a reduction in the risk of preeclampsia (1 to 3 appointments: *p* = 0.71, OR = 1.10; 4 or more appointments: *p* = 0.50, OR = 1.20). Thus, the presence or absence of prenatal care exerted no influence on the occurrence of preeclampsia.

Another case-control study^10^ conducted at a reference hospital involved a sample of 1,233 individuals: 650 in the case group (mean age: > 30 years) and 583 in the control group (mean age: 28 years). The prevalence of preeclampsia was not reported, but the condition was associated with fewer appointments (*p* < 0.001, OR = 2.04), which is in disagreement with the findings of another study in the present review.^12^

A prospective cohort study^16^ conducted at the National Department of Statistics analyzed 387,000 women over a 10-year period. Prenatal care was based on the implantation of a program aimed at controlling obstetric and psychosocial risk factors. The prevalence of preeclampsia ranged from 0.4 to 1.4% among women monitored at a private healthcare service (mean age: 24.2 ± 6.5 years) and 1.4 to 3.2% among women with no access to a private healthcare service (mean age: 25.1 ± 6.9 years). The effect of prenatal care involving the biopsychosocial model reduced the incidence of preeclampsia by 22% (OR = 0.78, 95% CI: 0.67–0.88), which is in agreement with the findings of another study,^10^ suggesting that an increase in prenatal care reduces the risk of PIH.

A retrospective study^14^ conducted at university hospitals involved a sample of 1,015 individuals: 612 (60.3%) with preeclampsia, 346 (34.1%) with eclampsia and 57 (5.6%) with another type of PIH. The mean age was 25.8 years. A lack of prenatal care increased the risk of the emergence of PIH (OR 2.3; 95% CI: 1.19–4.38), which is in agreement with the finding of 2 other studies.^10^ ^16^

A case-control study^11^ conducted at 3 reference hospitals involved 299 women: 80 in the case group (prevalence of chronic hypertension: 52%) and 219 in the control group (prevalence of chronic hypertension: 47%). The ages ranged from 15 to 49 years, with a mean of 29.2 years in the case group and 28.4 in the control group. A lack of prenatal care increased the risk of PIH (preeclampsia and eclampsia as well as preeclampsia and eclampsia complicated by hemorrhage) (OR = 3.97; 95% CI: 1.42–11.09). Therefore, the absence of prenatal care was related to the occurrence of different types of PIH, placing patients at risk.

In a cross-sectional study^15^ conducted at 5 public maternity hospitals involving a sample of 7,325 women (55.4% aged 25–34 years, 29.7% aged 24 years or younger and 14.9% aged 35 years or older), the prevalence of PIH (gestational hypertension, preeclampsia and eclampsia) was 3.19%. Prenatal care was not associated with PIH, as a larger or smaller number of appointments at public or private services did not exert an influence on the emergence of PIH (low-risk pregnancy: OR = 1.12; high-risk pregnancy: OR = 0.53), which is in agreement with the findings of another study in the present review.^12^

In a prospective cohort study^13^ conducted at maternity care clinics involving a sample of 10,314 women with a mean age of 66.7 years, the prevalence of PIH was 17%. Prenatal care was associated with the outcome, as women with ISH, IDH, DHP, GH and CH had a larger number of prenatal appointments than normotensive women, which is in agreement with findings reported in two other studies,^12^ ^15^ suggesting that prenatal care does not exert an influence on the reduction in or emergence of PIH.

Studies with a sufficient follow-up period involving women of different ages and with the control of possible confounding factors are needed to confirm the effect of prenatal care on the occurrence of PIH. The present review was conducted following the preferred reporting items for systematic reviews and meta-analyses (PRISMA) checklist.^19^

## Conclusion

The present findings demonstrate the importance of greater prenatal care as a measure for health promotion and a reduction in the occurrence of pregnancy-induced hypertension. Standardized methods are needed to strengthen the statistical power of the studies and prospective investigations are needed to gain a better understanding of the association between these two variables.
